# International Society for Computational Biology Honors Michael Ashburner and Olga Troyanskaya with Top Bioinformatics/Computational Biology Awards for 2011

**DOI:** 10.1371/journal.pcbi.1002081

**Published:** 2011-06-02

**Authors:** Justin Mullins, BJ Morrison McKay

**Affiliations:** 1 Freelance Science Writer, London, United Kingdom; 2 International Society for Computational Biology (ISCB), University of California San Diego, La Jolla, California, United States of America

## Introduction

Each year, the International Society for Computational Biology (ISCB; http://www.iscb.org/) makes awards for exceptional achievement to two scientists. The first is presented to a scientist who has made distinguished contributions over many years in research, teaching, service, or any combination of the three. This year, the ISCB Accomplishment by a Senior Scientist Award goes to Michael Ashburner in the department of genetics at the University of Cambridge. The second, known as the Overton Prize, honours a young scientist in the early to mid-stage of his or her career who has already achieved significant and lasting impact in the field of computational biology. In 2011, the Overton Prize is awarded to Olga Troyanskaya of Princeton University in New Jersey.

The recipients were chosen by the ISCB's awards committee chaired by Alfonso Valencia at the CNIO (Spanish National Cancer Research Centre) in Madrid. The winners will receive their awards at the ISCB's annual meeting, where they will also deliver keynote talks. This meeting, ISMB/ECCB 2011 http://www.iscb.org/ismbeccb2011, will take place in Vienna, Austria, 17–19 July 2011.

## 2011 Accomplishment by a Senior Scientist Award: Michael Ashburner

**Image 1:**
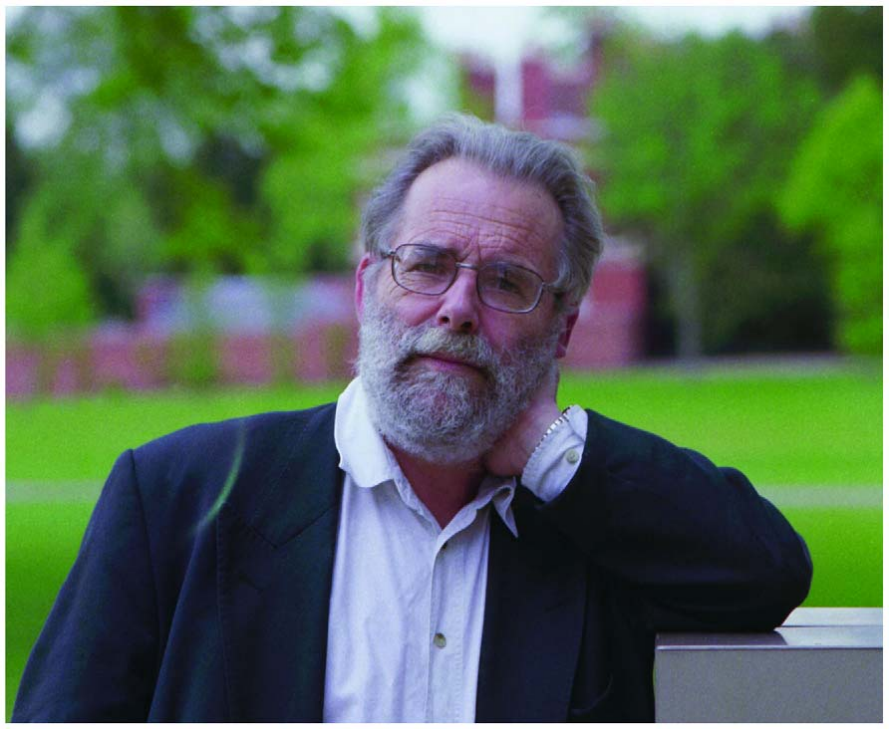
Michael Ashburner. Photo courtesy of European Molecular Biology Laboratory.

If computational biology seems challenging in the second decade of the 21st century, spare a thought for those who pioneered the discipline in the 1980s. Michael Ashburner (Image 1) at the University of Cambridge was one of them. “His work is now seen as a landmark and an achievement in technology,” says Alfonso Valencia, chair of the ISCB awards committee.

Ashburner began his career with a degree in genetics from the University of Cambridge in 1964. He stayed on to do a PhD, studying *Drosophila* and, in particular, polytene chromosomes, which form when certain specialised cells undergo repeated rounds of DNA replication. Polytene chromosomes have a characteristic banded structure. In *Drosophila* there are some 5,000 bands and a subset of these undergo, during development, a reversible structural modification as the result of transcription; this is know as puffing and can be considered an analog of gene activity. In the late 1960s and early 1970s, Ashburner studied puffing patterns and inferred the existence of a cascade of genetic controls under the influence of the hormone ecdysone during larval development.

In the late 1970s, Ashburner turned his attention to the study of the *Alcohol dehydrogenase* gene and its environs. By the mid-1980s, he had the most detailed analysis in full genetic terms of any small chromosome region of any multi-cellular organism, and had the *Adh* gene sequences from several different species of *Drosophila*. “That drew me into bioinformatics because we needed a way of comparing sequences,” he says. “There was almost no software available to help.”

Two people came to his aid. The first was Walter Bodmer, director of the Imperial Cancer Research Fund, who gave Ashburner the use of a DEC computer with access to the early network. “We could access this machine by dial-up and do some analysis,” he says. The second was Doug Brutlag at Stanford University, who was developing MOLGEN, an early bioinformatics system, which he allowed Ashburner to access.

That presented a significant obstacle, however. Getting a computer in the United Kingdom to speak to one in Stanford was not straightforward. Today, everybody uses the Internet, defined by the TCP/IP protocol. But in the early ‘80 s, the UK and United States used different systems. The US was pioneering TCP/IP while the UK had a standard called the Coloured Book protocols. “The only place that had an interface between the two protocols was University College, London, and they were very helpful,” says Ashburner, “giving us 5 kb of disk space.”

The process of connecting to Stanford was far from simple. “The way you did it was to dial up your local packet switching exchange at the Post Office and connect to the Rutherford Appleton Laboratory. You then typed in some code which connected you to UCL where you could use TCP/IP,” he says. The signal was routed via Goonhilly satellite station in Cornwall to Carnegie Mellon University and from there to Stanford. “I had a dumb terminal, that is a box with no memory, so everything had to be captured by a printer in parallel.” Ashburner was far from deterred, however.

At about that time, the European Molecular Biology Laboratory (EMBL) in Heidelberg and GenBank in the US released the first nucleotide sequence libraries in quick succession. Using his network access, Ashburner and his colleagues, collaboratively with MOLGEN, set up one of the first bulletin boards, called BioNet, to keep people informed of changes to the library and to software. “This became well used and things evolved from there,” he says.

As the field of bioinformatics grew, the need for an institution to house the data and conduct research increased. So in 1992, the EMBL decided to set up an institute of bioinformatics that would house this library and carry out research. This organisation became known as the European Bioinformatics Institute, based in Hinxton, UK, with Ashburner and John Sulston having led the UK bid to host it. “I was persuaded to become the first program coordinator and took half-time leave from Cambridge to do that,” he says. He eventually took over as joint-director, a post he held until 2001. “At first, the finances were sticky and the politics were horrendous. But it has since gone from strength to strength,” he says.

At the same time, Ashburner continued his interest in *Drosophila* genetics. This is a field with a rich and long history of collecting and sharing mutations. The first catalogue of mutations was published in 1925 and it was still being revised in paper form in the late 1980s. But the field was beginning to expand quickly and the books were out of date as soon as they were published. “It became clear to me that we couldn't carry on publishing in paper form every 10 or 20 years,” he recalls.

So in 1989 he proposed that the community set up an electronic database to take over the role of the printed one. In 1992, the NIH funded the project that became known as FlyBase, one of the first genetic and now genomic databases.

FlyBase was a crucial factor in triggering Ashburner's interest in a structured, controlled vocabulary, a formal representation of knowledge about genes and gene products. He began to define terms for gene products by their biological processes, such as wing development, and then defined the data structure in which these terms were related to each other. “It occurred to me that if you were able to do this for several model species, you'd have a fantastic tool,” he says.

But this insight initially met with little interest. “My first presentation, at ISMB in Greece in 1997, went down like a lead balloon,” he recalls. Eventually, he and three like-minded colleagues settled the matter in a bar at the Montreal ISMB in 1998. Together, they decided to set up a cross-species ontology to be used by the *Drosophila*, yeast, and mouse databases. They called it the Gene Ontology, and it is now a major bioinformatics project that covers over 1,800 species. Their original paper on the idea in *Nature Genetics* is one of the most highly cited in the field. “His achievement is not just to have built this system but also to have organised the consortium behind it. It is now one of the most used resources in all of biology,” says Valencia.

He went on to collaborate with Gerry Rubin and Craig Venter in sequencing the *Drosophila* genome in 1999. “The process turned me into a nervous wreck,” he jokes. He published his account of this roller-coaster experience in a short but entertaining book called *Won for All: How the Drosophila Genome was Sequenced* (Cold Spring Harbor Laboratory Press, 2006).

“We're lucky to have such an inspirational figure in the community,” says Valencia. “This award has been well deserved for a number of years.”

## 2011 Overton Prize: Olga Troyanskaya

**Image 2:**
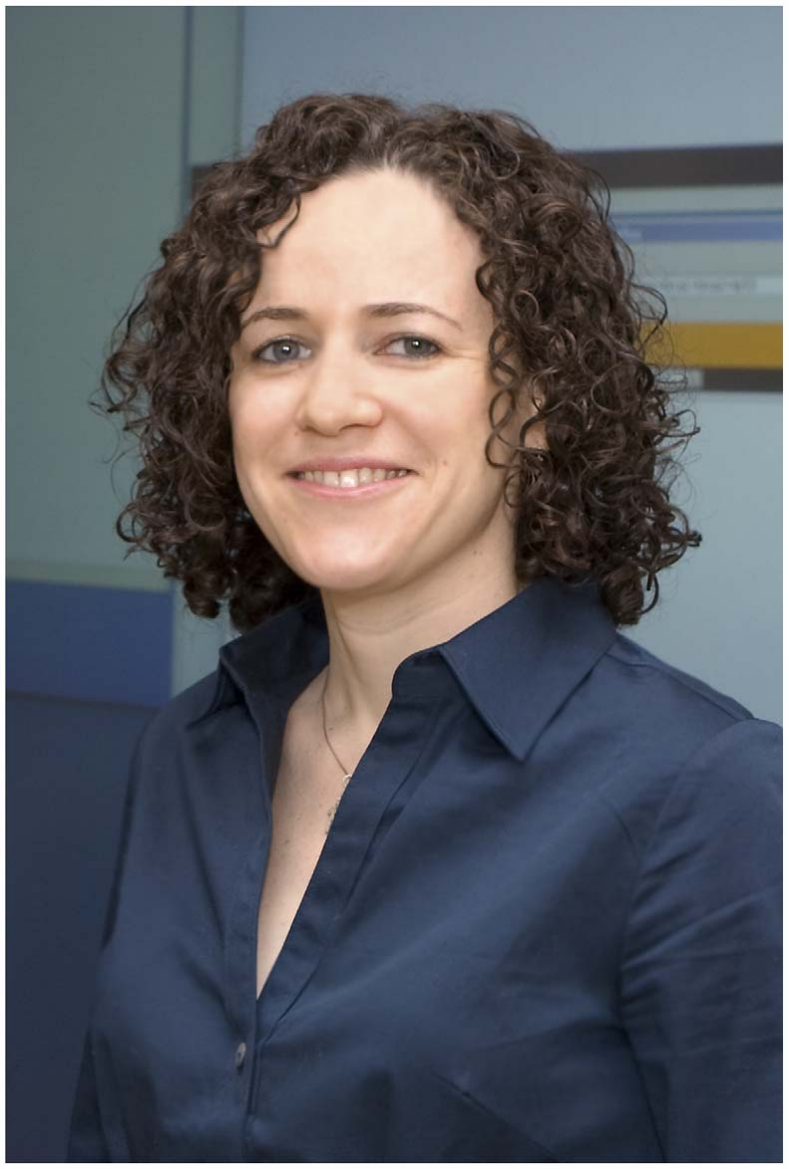
Olga Troyanskaya. Photo courtesy of Princeton University, Office of Communications.

In the spring of 1997, Olga Troyanskaya (Image 2) was working on a degree in computer science and biology at the University of Richmond, Virginia, when she contacted Steven Salzberg, then at Johns Hopkins University, about a summer internship in his lab devoted to computational biology. “He took a chance on me—a random student from another school—and was tremendously inspirational,” she says. She spent the following two summers working in Steven Salzberg's laboratory, first at Johns Hopkins and then at The Institute for Genomic Research.

And so began the career of one of the most promising young researchers in bioinformatics, and a deserving winner of this year's Overton Prize. “She is one of these forces of nature, full of energy,” says Alfonso Valencia, chair of the ISCB awards committee.

Troyanskaya herself talks with infectious enthusiasm about her work. “I've always been fascinated by the problems of biology,” she says. “I was just better at computer science and math than the wet lab research. And it seemed to me that there had to be a lot you could contribute with computer science that you couldn't do with experimental techniques alone.”

From the University of Richmond, Troyanskaya moved to Stanford University to complete a PhD in biomedical informatics, under the supervision of Russ Altman, a bioinformatician, and David Botstein, a geneticist. “I wanted a setup that was close to real biological problems, and I got exactly that. I learned a great deal from both of them,” she says.

In 2003, she moved to Princeton University as an assistant professor in the Department of Computer Science and the Lewis-Sigler Institute for Integrative Genomics. “I am fortunate that the computer science department appreciates the impact of computing in biology, and that I have many wonderful colleagues at both the department and in the Institute. I found several amazing collaborators, and this allowed me to begin a number of interesting projects.”

One of the key problems she focuses on is making better use of the vast but unwieldy biological datasets in databases around the world. “So instead of focusing on one study, we can take the entirety of published data. That allows you to ask very specific questions in a data-driven way and to develop novel biological hypotheses,” she says.

An important goal is to predict the function of genes or proteins. There have been many experimental approaches to determine what genes do and how they are controlled inside the cell. But this work tends to produce datasets that are large and noisy. Troyanskaya's approach is to develop new ways for extracting useful information from these datasets using techniques from computer science such as machine learning and data mining.

“Computation by itself is often not enough to discover new biology but it can direct experimental work,” she says. And she has set up a wet lab to help test and validate the hypotheses that the computer science helps generate. In 2009, for example, she used this approach to identify 109 new proteins involved in mitochondrial biogenesis in yeast.

This combined approach is one of the things that sets Troyanskaya apart, says Valencia. “She is one of the first to have come from the computational side and then moved into the experimental area to combine both,” he says.

Understanding the function of individual genes is only a small part of a much bigger story. Many genes and proteins play multiple roles within a cell as parts of various networks of biological processes. Mapping out these networks and understanding how they work and interact with each other is yet another strand of her research. “She has made important contributions to systems biology,” says Valencia.

The process of evaluating and validating computational predictions is an area requiring a broad collaboration to develop standards and methods that can be used to achieve a consensus about the results. To this end, Troyanskaya is collaborating with the curators of model organism databases and members of the Gene Ontology Consortium.

Another problem that many researchers face is handling the data avalanches currently being generated. So Troyanskaya, in collaboration with Princeton colleagues Kai Li and Moses Charikar, is looking at ways to better search and visualise these huge datasets, something that is challenging because of high noise levels and the enormous volume of the data. “We are developing better ways to do this,” she says.

The awards committee was also impressed by Troyanskaya's service for the community. She is involved in the Society's two official journals, *PLoS Computational Biology* and *Bioinformatics*. And she is involved in conferences: organizing, chairing tracks and program committees. “That is something that is very much appreciated,” says Valencia. “We are lucky to have her.”

And there is surely more to come. Troyanskaya points to numerous questions that are driving her research forward. She wants to know, for example, how we can predict which genes are involved in kidney disease, to understand their function and their clinical role on a molecular level. She works on these questions in close collaboration with experimental researchers, such as Matthias Kretzler and his group from the University of Michigan, Ann Arbor. And she is passionate about finding ways to ask questions in a data-driven way, not just in a knowledge-driven way that relies on what we already know about biology. “These are the questions that I'm really interested in,” she says. “And we really haven't yet harnessed the full potential of our data collections.”

## Additional Information

The full conference agenda and registration information for ISMB/ECCB 2011, where these ISCB award winners, along with four other distinguished Keynote lecturers, can be found on the conference Web site at http://www.iscb.org/ismbeccb2011. The conference will also feature parallel tracks for Proceedings of original research papers, Highlights of recently published papers, Special Sessions on emerging topics, Late Breaking Research of peer-reviewed abstract submissions, and Technology demonstrations and workshops presented by academic researchers and commercial vendors. The conference also displays a unique “Art and Science” exhibit of scientifically based artistic visual images and videos submitted, and offers a commercial and non-profit vendor exhibition.

For a review of past ISCB award winners, please see http://www.iscb.org/iscb-awards.

